# HydroBot: Software for Interactive Hydrogen/Deuterium-Exchange Mass Spectrometry Multistate Analysis

**DOI:** 10.1021/jasms.5c00311

**Published:** 2025-12-18

**Authors:** Monika Kish, Jonathan J. Phillips

**Affiliations:** 1 Living Systems Institute, 3286 University of Exeter, Stocker Road, Exeter EX4 4QD, U.K.; 2 Department of Biosciences, 3286 University of Exeter, Stocker Road, Exeter EX4 4QD, U.K.

## Abstract

Hydrogen/deuterium-exchange mass spectrometry (HDX-MS) is a powerful technique for probing protein dynamics, stability, and interactions. However, multistate and nonequilibrium experiments currently do not have available analysis tools. We present HydroBot, a software designed for comprehensive and interactive HDX-MS data analysis and visualization. HydroBot supports rapid and automated uptake plotting, statistical testing of differences between protein states, and multiple interactive visualization modes including bar plots, Woods plots, and heatmaps. Statistical tools such as volcano plots and error distribution analyses are integrated to assess data robustness, enabling interactive exploration of labeling differences. Correlated structural dynamics are revealed by *k*-means or hierarchical clustering, facilitating pattern discovery within multistate HDX data for proteins at equilibrium and nonequilibrium. Additionally, scripts are created to enable visualization of HydroBot analyses on protein structures. This user-friendly tool streamlines HDX-MS validation and interpretation from processed data to biologically relevant insights.

## Graphical abstract

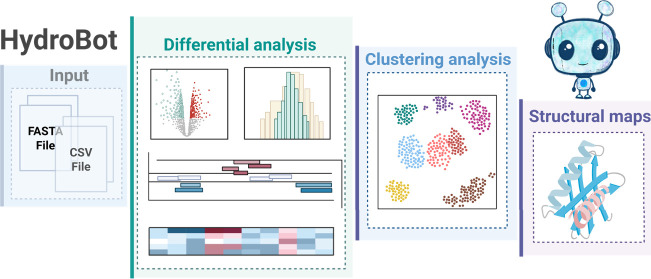

## Introduction

Hydrogen/deuterium-exchange mass spectrometry (HDX-MS) has emerged as a pivotal technique for investigating protein conformational dynamics. By measuring the exchange rates of backbone amide hydrogens with deuterium,^1^ HDX-MS provides submolecular insights into protein flexibility and structural changes in almost any formulation^2^ or physiological condition.^3^

Interpretation of correlated changes, such as during allostery, from HDX-MS data remains challenging due to the complexity of the data sets and the need for robust statistical validation with visual inspection to arrive at the optimal analysis. Current HDX-MS software offers valuable solutions for aspects of the analysis workflow, including HD-eXplosion,^4^ Deuteros,^5^ HaDeX,^6^ HDX-Viewer,^7^ HDXboxeR,^8^ HDFlex,^9^ PyHDX,^10^ MEMHDX,^11^ and HDGraphiX.^12^ However, there remains a need for fully interactive plotting of data following robust statistical analysis and clustering to identify correlated structural dynamics, especially for multistate analysis. To address these gaps, we developed HydroBot, an interactive software application that uniquely enables the structural mapping of correlated structural dynamics alongside standard statistical analysis. Beyond integrative interactivity, HydroBot introduces several unique features, including three-state coordinate construction for clustering and peptide trajectory visualization. By combining these functionalities in an easy-to-use, open framework, HydroBot provides a streamlined environment for rapid and reproducible interpretation of complex HDX-MS data sets, including multistate data.

## Materials and Methods

### Statistical Analysis

To assess pairwise differences between protein states, HydroBot performs statistical tests including Welch’s *t* test^13^ and applies a global significance threshold^14^ to control false discovery rates. Global significance thresholds (GSTs) are computed from the pooled standard deviation, converted to a standard error of the mean for the two states, and multiplied by the two-sided *t*-critical value at the chosen alpha; we report this value as the GST (details in the Supporting Information). A peptide is considered significant only if it meets both criteria: (i) its uptake difference exceeds the global threshold, and (ii) the Welch’s *t* test *p*-value is below the selected α level. Volcano plots^15^ are used to visualize statistically significant uptake differences against the magnitude of change. Error distributions are given as frequency histograms to evaluate replicate variability and confidence in the measured uptake values.^9^ This allows the user to query data reliability and experimental reproducibility and facilitates the identification of outliers and potentially their removal.

### Clustering

HydroBot implements unsupervised *k*-means and hierarchical clustering to identify correlated behaviors in peptides or amino acids based on similarity in their HDX kinetics. Silhouette and elbow plots help optimize the cluster number, and uptake data can be converted into scalar values (e.g., sum difference) for [*x*,*y*] clustering coordinates.

### Software Availability

HydroBot, documentation, and example data sets are available open-source (GitHub-monikakish/HydroBot).

## Results and Discussion

### Statistical Analysis and Visualization

Users can first generate deuterium uptake plots for all of the available protein states. Plots are displayed interactively, allowing visualization of uptake trends with values normalized to the theoretical maximum deuterium uptake (MaxD) per peptide.

Global significance threshold (GST) is then calculated for pairwise combinations similarly to methods explained elsewhere,^14^ enabling the assessment of meaningful differences between conditions. Users can also input a GST calculated elsewhere.

Differential uptake is then computed per peptide per time point and per residue per time point (linear weighting similar to Keppel and Weis; Figure S1),^16^ and then a sum of these uptake differences is calculated. Interactive volcano plots display uptake differences (Δ*D*) between two states on the *x*-axis and their statistical significance (−log_1__0_ *p*-value, Welch’s *t* test) on the *y*-axis (Figure 1B).

**Figure 1:**
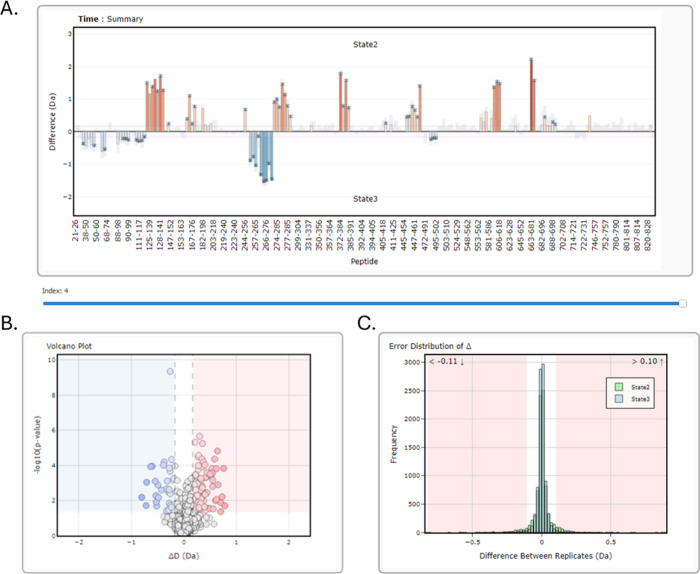
HydroBot statistical significance outputs. (A) Difference bar plot between two states per peptide, colored on a gradient from blue (lowest significantly different values) to red (highest significantly different values) across the data set. Asterisks indicate statistically significant differences at the chosen *p*-value α threshold. The gray shaded region shows the limit of statistical significance. (B) Volcano plot displaying uptake differences between the two states, with the global significance threshold indicated by the shaded region. Differences identified by hybrid significance testing are highlighted in blue (relative protection) or red (relative deprotection). (C) Error distribution of replicate measurements in both states, illustrating variability and confidence in the data and aiding in outlier identification.

Error distributions are calculated per peptide per state by using a bootstrapping approach. For each peptide at each exposure time, pairwise replicate differences are sampled to generate bootstrap estimates of the mean difference. The combined bootstrap means across all peptides form the overall error distribution from which 95% confidence intervals are derived. Values outside these intervals (red shaded regions) are considered significant. Histograms are plotted separately for each state, with hover information showing the state and peptide sequence (Figure 1C).

Users can choose between a bar chart or a Woods plot^17^ for visualizing peptide-level differences (Figure 1A). In a Woods plot, the *x*-axis represents the peptide position in the protein sequence and the *y*-axis shows the difference in uptake between states. Furthermore, Woods plots provide direct visualization of peptide overlap and sequence coverage.

As an interactive tool, HydroBot allows users to inspect the volcano and error distribution plots and remove peptides identified as outliers, refining the data set for further analysis.

For amino acid-level analysis, differential uptake is calculated by averaging the contributions of all overlapping peptides covering each residue.^16^ This aggregation allows the identification of site-specific changes in deuterium incorporation that may be masked at the peptide level. The resulting per-residue differences can be visualized as bar charts or heatmaps, complementing peptide-level volcano and Woods plots by providing higher-resolution insight into local conformational dynamics.

### Clustering Analysis

The clustering tab in HydroBot allows users to explore patterns of differential deuterium uptake across peptides, residues, or time points between three states. Notably, this is particularly important in order to reveal transient correlated changes for nonequilibrium experimental data where there are equilibrium data available for valid reference states, such as the start/end states.^18^ Both *k*-means and hierarchical clustering methods are implemented, enabling the identification of groups of peptides that exhibit correlated dynamics or conformational changes.

Clustering is based on a three-state analysis, where the D-labeling data for one state is subtracted per time point from two reference states for each peptide segment, resulting in an [*n* × 2] vector of 2D coordinates. The clusters are evaluated at each mixing time and for sum uptake. Peptide-level data are then flattened per residue, as previously described.^16^ These 2D vectors provide well-formed input for clustering and facilitate an interactive analysis of scatter plots (Figure 2A).

**Figure 2:**
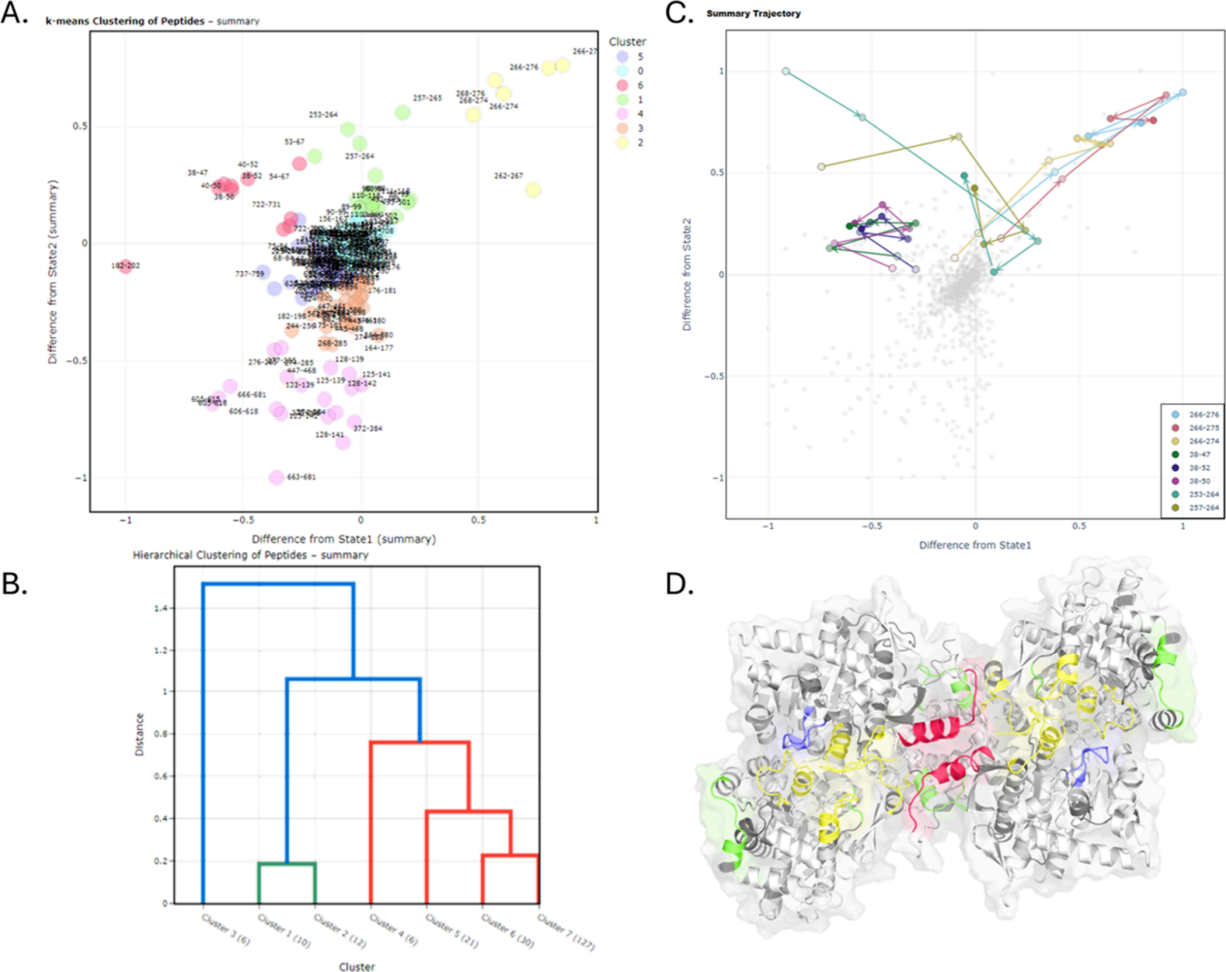
Clustering analysis outputs were from HydroBot. (A) *k*-means scatter plot of peptides grouped by similar differential deuterium uptake (Δ*D*) dynamics. (B) Hierarchical clustering dendrogram showing relationships and similarities between peptide clusters. (C) Peptide-level Δ*D* trajectories across labeling time points, illustrating dynamic changes over time. (D) Example of Cluster 1,2,6 mapped onto the protein structure using PyMOL.

In *k*-means clustering, peptides with similar Δ*D* relative to the two reference states are grouped into clusters, with a user-defined number of clusters, determined with input from the elbow and silhouette analyses. Hierarchical clustering provides an unsupervised method to identify correlated peptides, which is done using Ward’s linkage and Euclidean distance, generating a dendrogram that visualizes the relationships between peptide clusters. This approach allows users to examine both the hierarchy and similarity of peptide dynamics over time, complementing the insights provided by *k*-means clustering (Figure 2B).

### Structural Visualization

Structural resolution of correlated changes between protein states is frequently insightful to infer mechanistically important features. HydroBot exports correlated clusters for 3d visualization on a PDB model (Figure 2D). Clusters generated are exported as PyMOL script (.pml).

### Peptide Trajectory

The Peptide Trajectory tab visualizes the temporal evolution of peptides across clusters identified by *k*-means analysis. Each trajectory plot tracks a single peptide, showing how its cluster membership changes over successive D_2_O labeling time points, and overlaying trajectories from multiple protein peptides to directly compare dynamic behavior (Figure 2C). This feature is immediately useful to discover intermediates from nonequilibrium HDX-MS data, such as during allostery, catalysis, redox, and biopharmaceutical long-term stability.

By linking trajectory data with peptide-level differential analysis and clustering results, users can track dynamic events across the protein and identify peptides that switch clusters over time, enabling identification of unique conformations from allosteric effects, or local unfolding events—even when these are present only transiently.

## Conclusions

Here, we present HydroBot, an interactive software application that uniquely enables structural mapping of correlated structural dynamics alongside commonly sought robust statistical analysis in a single environment, interpreting by enabling interactive visualization of multistate and correlated dynamics. By combining established analyses with intuitive interactivity and publication-ready outputs, HydroBot stands to provide a valuable and accessible resource for exploring protein dynamics through HDX-MS.

## Supplementary Material



## Notes

Software design and implementation; data input and preprocessing; uptake plots; global significance threshold calculation; volcano plot visualization; error distribution and bootstrap estimation of replicate variability; residue level averaging; preprocessing for clustering; determination of optimal cluster number; and structural mapping in PyMOL (PDF)
