# Supplementary material for: HydroBot: Software for Interactive Hydrogen/Deuterium-Exchange Mass Spectrometry Multistate Analysis
Source: J Am Soc Mass Spectrom. 2025 Dec 18;37(1):341–5. doi: 10.1021/jasms.5c00311 (PMC12784399; doi:10.1021/jasms.5c00311)
Supplement: Supplementary file 1 [file js5c00311_si_001.pdf]

Supplementary information

HydroBot: Software for interactive hydrogen/deuterium-exchange mass spectrometry multi-state analysis

Monika Kish[*1,2], Jonathan J. Phillips[*1,2]

[1]Living Systems Institute, University of Exeter, Stocker Road, Exeter, EX4 4QD, UK

[2]Department of Biosciences, University of Exeter, Stocker Road, Exeter, EX4 4QD, UK

AUTHOR INFORMATION

Corresponding Authors

Jonathan J. Phillips − Living Systems Institute, University of Exeter, Exeter EX4 4QD, U.K.; Department of Biosciences, University of Exeter, Exeter EX4 4QD, U.K.; orcid.org/0000-0002-5361-9582

Email: jj.phillips@exeter.ac.uk

Monika Kish − Living Systems Institute, Department of Biosciences, University of Exeter, Exeter EX4 4QD, U.K.; orcid.org/0000-0002-3661-8641

Email: m.kish@exeter.ac.uk

Notes The authors declare no competing financial interest.

Software Design and Implementation. HydroBot is developed as an interactive desktop application using Python 3.11. Data processing and statistical analyses leverage pandas (2.1.3), NumPy (1.26.2), and SciPy (1.12.0), while dynamic visualizations are created with Plotly (6.1.0), matplotlib (3.8.2), and seaborn (0.12.2). The GUI is built with PyQt5 (5.15.11), enabling intuitive interaction and seamless navigation through the HDX-MS data analysis workflow. Clustering and statistical analyses utilize scikit-learn (1.2.2), supporting k-means and hierarchical clustering, as well as Welch's t-test for assessing uptake differences. The application allows users to import processed (typically centroided) HDX-MS datasets, process them automatically, visualize results with interactive plots (bar plots, heatmaps, Woods plots, volcano plots), and export both graphical outputs and processed data tables.

Data Input and Preprocessing. HydroBot is organized into four interactive tabs: HDX and Difference Analysis, HDX Data Clustering, Peptide Trajectory, and File Conversion. As input the application directly reads comma-separated value (CSV) files of cluster and state processed HDX data, in the format natively generated by DynamX. Alternatively, it will import and convert data in other formats, enabling conversion and analysis of datasets from virtually any HDX-MS processing software in an

agnostic manner. Users need to ensure that the centroided HDX data .csv file contains the necessary columns in the format expected by HydroBot. Sample input files are provided with the HydroBot distribution. A FASTA file with the protein sequence is also required, and an output folder must be chosen for exports.

Uptake plots. Uptake plots can be generated for any/all selected states with uptake values normalized to the peptide maximum uptake (100%). Plot colors and time units (ms, s, min) are customizable. Plots are interactively visualized within the application to aid exploration and interpretation.

Global significance threshold. The application calculates a global significance threshold for each selected pairwise combination of states using statistical metrics explained elsewhere[1]. Users can also input a global significance threshold calculated elsewhere. However the user chooses to generate a GST, HydroBot applies the value consistently across all calculations and plots.

Briefly to determine the Global Significance Threshold (GST) for differential uptake between two protein states HydroBot calculates a pooled standard deviation across peptides, weighting each peptide by its number of replicates, to more accurately represent variance in differential uptake. For each comparison between states A and B, the GST represents the minimum absolute difference in mean uptake required to be considered statistically significant at a given confidence level (α).

If n_A and n_B denote the number of replicates for states A and B, respectively, and s_A and s_B represent the uptake standard deviations across peptides for each state. The pooled standard deviation (s_p) is computed as:

$$s_p = \sqrt{\frac{(n_A - 1)\sum s_A^2 + (n_B - 1)\sum s_B^2}{(n_A - 1)N_A + (n_B - 1)N_B}}$$

Equation S 1

where N_A and N_B are the number of peptides quantified for each state.

The standard error of the mean difference between states is given by:

$$SEM = \sqrt{\frac{s_p^2}{n_A} + \frac{s_p^2}{n_B}}$$ Equation S 2

Assuming equal variances, the degrees of freedom are df $= n_A + n_B - 2$. The two-tailed Student's t critical value for significance level $\alpha$is then determined as:

$$t_{\alpha/2,\, df} = t_{ppf}(1 - \alpha/2, df)$$ Equation S 3

Finally, the Global Significance Threshold (GST) corresponding to the confidence interval for the difference between states is calculated as:

$$GST = t_{\alpha/2,\, df} \times SEM$$ Equation S 4

A default α of 0.05 (95% confidence) is applied, with user-selectable options for α = 0.10 or 0.01. Replicate counts (n_A,n_B) are automatically derived for each peptide from the dataset, allowing the Global Significance Threshold (GST) to account for variation in replicate number across peptides.

The resulting GST value is rounded to two decimal places and applied as a cutoff for identifying significant uptake differences. GST computation uses per-peptide replicate counts (i.e. from cluster data produced by e.g. DynamX), ensuring that peptides with fewer replicates contribute proportionally to the pooled variance and standard error. In contrast to the approach of Hageman & Weis (2019), uptake differences are evaluated directly, and the global significance threshold is calculated from the pooled standard deviation and t-critical value without additional standardization by standard error. HydroBot does not currently apply a formal multiple testing correction such as Benjamini–Hochberg to control false discovery rates.

Volcano plot visualization of differential uptake. To summarize and visualize differential uptake between two protein states, HydroBot generates volcano plots. Volcano plots display uptake differences (ΔD) between two states on the x-axis and their statistical significance ($-\log_{10}$ p-value, Welch's t-test) on the y-axis. Each point is a peptide/fragment, with hover text showing the residue range. Significant peptides are colored by ΔD (blue–white–red), non-significant ones in grey. Vertical dashed lines mark the GST, a horizontal line the p-value cutoff, and shaded regions highlight peptides exceeding these limits (Figure 1B). For each peptide p, the uptake difference between states 1 and 2 is calculated as:

$$\Delta D_p = D_p^{(S_1)} - D_p^{(S_2)}$$

Equation S 5

Statistical significance is represented as the negative log-transformed p-value:

$$y_p = -\log_{10}(p\text{-value}_p)$$

Equation S 6

Points are coloured according to the magnitude and direction of ΔD_pusing a blue-white-red gradient, while nonsignificant peptides ($p > \alpha$) are displayed in grey. Threshold lines indicate the global significance threshold (GST) along the x-axis and the chosen p-value cutoff αalong the y-axis. Regions exceeding these thresholds are shaded to guide interpretation.

Hover text displays peptide start–end positions and exposure times. The layout is optimized for clarity, including gridlines, mirrored axes, consistent fonts, and marker styling. This visualization allows users to quickly identify peptides with meaningful differential uptake while assessing both magnitude and significance in a single view.

Error distribution and bootstrap estimation of replicate variability. To assess experimental variability within each protein state, HydroBot estimates the error distribution of replicate uptake measurements using a nonparametric bootstrap approach. This analysis quantifies the distribution of pairwise replicate differences and determines confidence bounds for random variability.

For each peptide p and exposure time t within a given state S, all pairwise replicate uptake differences are computed as:

$$\Delta U_{i,j}^{(S,p,t)} = U_i^{(S,p,t)} - U_j^{(S,p,t)}, i \neq j$$

Equation S 7

where $U_i^{(S,p,t)}$ and $U_j^{(S,p,t)}$ represent individual replicate uptake values.

From these pairwise differences, bootstrap resampling with replacement is applied to obtain n bootstrap samples, each consisting of s randomly selected replicate differences. The mean difference of each bootstrap sample is then calculated as:

$$\grave{\Delta U}_b^{(S,p,t)} = \frac{1}{s}\sum_{k=1}^{s} \Delta U_{k,b}^{(S,p,t)}, b = 1,\ldots,n$$ Equation S 8

The collection of all bootstrap mean differences across peptides, time points, and states forms an empirical error distribution representing replicate variability. The 95% confidence interval bounds of this distribution are determined from the 2.5th and 97.5th percentiles:

$$CI_{95\%} = [P_{2.5}(\grave{\Delta U}),\ P_{97.5}(\grave{\Delta U})]$$

Equation S 9

Values lying outside this interval are interpreted as exceeding the expected range of replicate variability (highlighted in red in Figure 1C). Histograms are plotted separately for each state, with hover information displaying the state, peptide start - end positions, and exposure time.

By visualizing the bootstrap-derived error distribution, HydroBot provides an empirical benchmark for assessing whether observed uptake differences are likely due to genuine state-dependent effects rather than replicate variability. Importantly, this bootstrap analysis is used for diagnostic purposes only: it allows users to identify potential outlier peptides but does not alter the hybrid framework's uptake calculations. This approach supports and complements the calculation of the GST, ensuring that the statistical cutoff applied for differential uptake analysis reflects the underlying measurement precision across replicates.

Residue level averaging of differential uptake. To resolve localized differences in deuterium uptake at single-residue resolution, peptide-level uptake differences were converted to residue level averages using a weighted averaging approach. For each exposure time t, peptides spanning residues n_1 to n_2 were mapped onto the protein sequence, and the uptake difference between states was normalized by the number of exchangeable amides within that peptide (total residues minus prolines and the N-terminal residue).

For a given peptide p covering residues $r = n_1 + 1,\ldots,n_2$, the per-residue contribution was calculated as:

$$\delta_{r,p}^{(S_1,S_2)} = \frac{\Delta D_p^{(S_1,S_2)}}{N_p}$$ Equation S 10

where $\Delta D_p^{(S_1,S_2)}$ is the peptide-level uptake difference and N_p is the number of exchangeable amides in the peptide. If multiple peptides covered the same residue, a weighted average was computed based on the number of exchangeable sites per peptide:

$$\grave{\delta}_r^{(S_1,S_2)} = \frac{\sum_p w_p \ \delta_{r,p}^{(S_1,S_2)}}{\sum_p w_p}, \text{with } w_p = N_p$$

Equation S 11

This weighting scheme ensures that longer peptides contribute proportionally to the averaged uptake, while preventing oversampling of shorter overlapping fragments. Corresponding filtered uptake differences and p-values were processed analogously to generate per-residue maps of filtered uptake ($\grave{\delta}_{r,\mathrm{fil}}$) and significance ($\grave{p}_r$). Direct averaging of p-values was avoided since they are not linearly additive. Instead, the per-residue significance mask reflects whether any peptide covering that residue exceeded the significance threshold ($p < 0.05$).

Proline residues were excluded from averaging due to their nonexchangeable amide hydrogens. Final per-residue values were compiled into a unified data frame indexed by amino acid position and sequence identity, with visual outputs highlighting statistically significant residues using an asterisk notation.

This residue-level aggregation enables finer spatial localization of uptake changes and facilitates subsequent structure mapping and visualization in PyMOL.

Currently HydroBot does not support analysis of electron transfer dissociation (ETD) or electron capture dissociation (ECD) data.

	Peptide						
Amino Acid	0	1	2	3	4	5	Weighted Average
H							
L							
F							
R	$1/5^2$						
K	$1/5^2$						
E	$1/5^2$						
L	$1/5^2$						
R	$1/5^2$						
L							
H		$3/10^2$					
D		$3/10^2$					
N		$3/10^2$					
P							
V		$3/10^2$	$2/5^2$	$3/7^2$	$2/10^2$	$2/7^2$	
L		$3/10^2$	$2/5^2$	$3/7^2$	$2/10^2$	$2/7^2$	
L		$3/10^2$	$2/5^2$	$3/7^2$	$2/10^2$	$2/7^2$	
A		$3/10^2$	$2/5^2$	$3/7^2$	$2/10^2$	$2/7^2$	
A		$3/10^2$	$2/5^2$	$3/7^2$	$2/10^2$	$2/7^2$	
L		$3/10^2$		$3/7^2$	$2/10^2$	$2/7^2$	
P							
S		$3/10^2$		$3/7^2$	$2/10^2$	$2/7^2$	
S					$2/10^2$		
E					$2/10^2$		
A					$2/10^2$		

Figure S 1. Schematic illustrating the data-flattening workflow, adapted from Keppel & Weis (2014). Each colour corresponds to a distinct uptake, with blank cells indicating missing data and grey squares marking fast-exchanging N-terminal amides. The panel on the right displays the flattened output, generated by computing the weighted average uptake for each amino acid position (per row) as described in Equation 11.

Preprocessing for clustering. Prior to clustering, peptide uptake data were pre-processed to ensure consistency across selected protein states (example: Apo, Eq, and non-Eq) and exposure times. Peptides were first filtered to remove any manually excluded sequences and those missing data for any state or common time point.

For each peptide p, state s, and exposure time t, the uptake value $U_{p,s}(t)$ was extracted. To represent the total deuterium incorporation per peptide within each state, uptake values across all common time points were summed:

$$S_{p,s} = \sum_{t=1}^{N_t} U_{p,s}(t) \quad \text{Equation S 12}$$

where N_t is the number of shared exposure times.

Pairwise uptake differences were then computed specifically for the non-Eq vs Eq and non-Eq vs Apo comparisons summed and per time point:

$$\Delta U_p^{(\text{noneq}-\text{Apo})} = U_{p,\text{noneq}}^{\text{sum}} - U_{p,\text{Apo}}^{\text{sum}}, \quad \Delta U_p^{(\text{noneq}-\text{Eq})} = U_{p,\text{noneq}}^{\text{sum}} - U_{p,\text{Eq}}^{\text{sum}}.$$

$$\Delta U_p^{(\text{non-Eq, Eq})} = S_{p,\text{non-Eq}} - S_{p,\text{Eq}}$$

$$\Delta U_p^{(\text{non-Eq, Apo})} = S_{p,\text{non-Eq}} - S_{p,\text{Apo}}$$

Equation S 13

To enable direct comparison across peptides with different uptake ranges, pairwise differences were normalized using **max-absolute scaling**, preserving both the sign and relative magnitude of uptake changes while constraining all values to the range [-1, 1]:

$$\widetilde{\Delta U}_p^{(s_i,s_j)} = \frac{\Delta U_p^{(s_i,s_j)}}{\max\left(|\Delta U_p^{(s_i,s_j)}|\right)} \quad \text{Equation S 14}$$

Additionally, per-time-point normalized differences were computed for each peptide and time point:

$$\widetilde{\Delta U}_{p,t}^{(s_i,s_j)} = \frac{U_{p,s_i}(t) - U_{p,s_j}(t)}{\max_t |U_{p,s_i}(t) - U_{p,s_j}(t)|} \quad \text{Equation S 15}$$

At the residue level, uptake differences were averaged per amino acid as described previously, by distributing each peptide's normalized uptake value across its covered residues and computing a weighted mean based on peptide length and the number of exchangeable residues.

Determination of optimal cluster number. *KMeans* clustering was applied to the per-peptide/residue normalized differences between non-equilibrium and Apo/Equilibrium states ($\widetilde{\Delta U}_p^{(\text{noneq}-\text{Apo})}$, $\widetilde{\Delta U}_p^{(\text{noneq}-\text{Eq})}$) to identify groups of peptides or residues exhibiting similar uptake changes.

To determine the optimal number of clusters (k), two complementary approaches were used:

Elbow method: This method evaluates the within-cluster sum of squares (WCSS) for a range of k:

$$\text{WCSS} = \sum_{i=1}^{k} \sum_{j=1}^{n_i} \text{distance}(x_j^{(i)}, c_i)^2 \quad \text{Equation S 16}$$

where:

$x_j^{(i)}$ is the j-th data point in cluster i,

c_i is the centroid of cluster i,

distance$(x_j^{(i)}, c_i)$ is the Euclidean distance between the point and its cluster centroid,

n_i is the number of points in cluster i,

k is the total number of clusters.

The WCSS measures how compact each cluster is. In the elbow plot, the "elbow" occurs where adding more clusters only slightly reduces WCSS, indicating the optimal k. In the elbow plot, the point at which the WCSS curve shows a clear bend (the "elbow") suggests the optimal k. If the curve flattens gradually, the elbow is taken where reductions in WCSS become minimal.

Silhouette analysis: The silhouette coefficient for each point $i$is defined as:

$$s(i) = \frac{b(i) - a(i)}{\max\,(a(i), b(i))},$$

Equation S 17

where $a(i)$ is the average intra-cluster distance and $b(i)$ is the minimum inter-cluster distance to any other cluster. Average silhouette scores across all points provide a measure of cluster cohesion and separation, with higher values indicating more distinct clusters.

In the silhouette plots, the number of clusters corresponding to the highest average silhouette score is considered optimal, indicating well-separated and coherent clusters. Individual silhouette plots for each k allow assessment of cluster quality and identification of potential outliers or poorly assigned points.

By combining these two metrics, a robust choice of k can be made, ensuring that clusters represent meaningful groups of residues or peptides with similar responses to non-equilibrium perturbations.

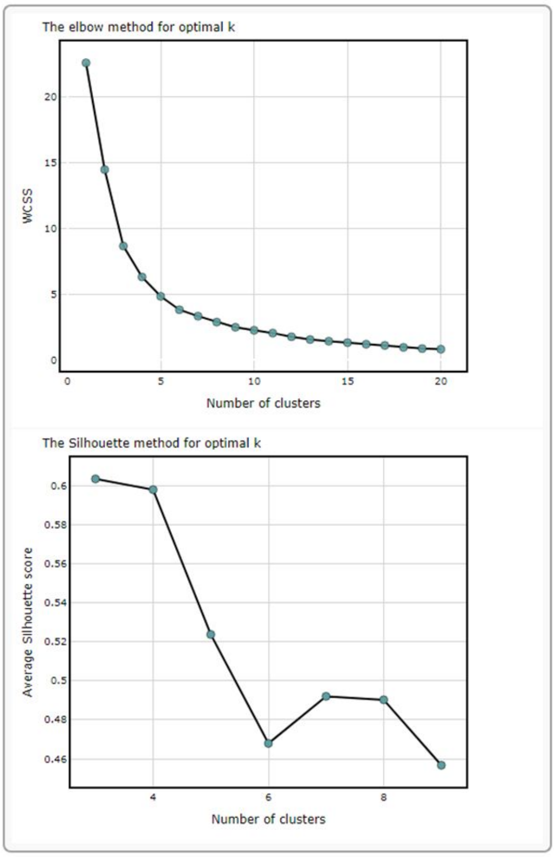

Figure S 2. Elbow and silhouette analysis for *k-means* clustering. Elbow plot (top) shows the within-cluster sum of squares (WCSS) as a function of cluster number (k); the optimal k is indicated by the point where WCSS decrease slows. Silhouette plot (bottom) shows average silhouette scores for each k, with higher values indicating more coherent and well-separated clusters, supporting selection of the optimal cluster number.

Structural Mapping in PyMOL. An enabling feature of HydroBot is the export of processed uptake data and identified clusters of correlated dynamics for direct visualization in PyMOL. Users can map uptake values or statistically significant differences onto protein structures (PDB files), represented as color gradients. HydroBot is compatible with legacy PyMOL free to use, owing to use of b-factors to deliver HDX data to the structure.

(1) Hageman, T. S.; Weis, D. D. Reliable Identification of Significant Differences in Differential Hydrogen Exchange-Mass Spectrometry Measurements Using a Hybrid Significance Testing Approach. *Anal Chem* **2019**, *91* (13), 8008-8016. DOI: 10.1021/acs.analchem.9b01325

(2) Woods, V. L., Jr.; Hamuro, Y. High resolution, high-throughput amide deuterium exchange-mass spectrometry (DXMS) determination of protein binding site structure and dynamics: utility in pharmaceutical design. *J Cell Biochem Suppl* **2001**, *Suppl 37*, 89-98. DOI: 10.1002/jcb.10069

(3) Keppel, T. R.; Weis, D. D. Mapping residual structure in intrinsically disordered proteins at residue resolution using millisecond hydrogen/deuterium exchange and residue averaging. *J Am Soc Mass Spectrom* **2015**, *26* (4), 547-554. DOI: 10.1007/s13361-014-1033-6

(4) Kish, M.; Ivory, D. P.; Phillips, J. J. Transient Structural Dynamics of Glycogen Phosphorylase from Nonequilibrium Hydrogen/Deuterium-Exchange Mass Spectrometry. *J Am Chem Soc* **2023**. DOI: 10.1021/jacs.3c08934
